# Almond Skin Inhibits HSV-2 Replication in Peripheral Blood Mononuclear Cells by Modulating the Cytokine Network

**DOI:** 10.3390/molecules20058816

**Published:** 2015-05-15

**Authors:** Adriana Arena, Carlo Bisignano, Giovanna Stassi, Angela Filocamo, Giuseppina Mandalari

**Affiliations:** 1 Department of Human Pathology, Policlinico Universitario, Via C. Valeria, Messina 98125, Italy; E-Mails: aarena@unime.it (A.A.); gstassi@unime.it (G.S.); 2 Department of Biological and Environmental Science, University of Messina, Sal. Sperone 31, Messina 98100, Italy; E-Mail: cbisignano@unime.it; 3 Department of Drug Science and Products for Health, Vill. SS. Annunziata, Messina 98100, Italy; E-Mail: afilocamo@unime.it

**Keywords:** HSV-2, cytokines, IL-17, almond skins

## Abstract

We have investigated the effect of almond skin extracts on the production of pro-inflammatory and anti-inflammatory cytokines in human peripheral blood mononuclear cells (PBMCs). PBMCs were either infected or not by herpes simplex virus type 2 (HSV-2), with and without prior treatment with almond skin extracts. Production of IL-17 induced by HSV-2 was inhibited by natural skins (NS) treatment. NS triggered PBMC in releasing IFN-α, IFN-γ and IL-4 in cellular supernatants. These results may explain the antiviral potential of almond skins.

## 1. Introduction

Defining the interactions of different immune cells involved in the response against infectious diseases is crucial to understand the complex network of host-microbe interactions in human pathology. We previously demonstrated that almond skins improve the immune surveillance of peripheral blood mononuclear cells (PBMCs) infected with herpes simplex virus type 2 (HSV-2) by triggering both the Th1 (IFN-α, IL-12, IFN-γ, TNF-α) and the Th2 (IL-4 and IL-10) response [1]. Moreover, NS treatment hinder the HSV-2 replication in PBMCs but not in WISH cell line, indicating that cell-mediated immunity was involved in the antiviral activity.

Polyphenols present in natural almond skin (NS) and its extracts post *in vitro* gastric and gastric plus duodenal digestion have been previously characterized [2,3], and are thought to modulate the immune response [1]. It has also been demonstrated that IFN-α inhibits production of IL-17 by PBMCs [4]. Recently, IL-17 has been described for its effect on hepatitis B virus replication and pathogenesis of liver injury in infected patients [5]. On the contrary, other authors have demonstrated that IL-17 enhances viral persistence [6]. In order to further understand the immunomodulatory effect of almond skins, here we investigate the production of IL-17, in PBMCs either infected or not by HSV-2. In addition, we analysed the immunoregulatory role of IFN-α, IFN-γ and IL-4 on IL-17 release.

## 2. Results and Discussion

Table 1 reports the cytotoxicity results as mean (%) of three independent assays. Non-cytotoxic concentrations were selected for subsequent assays.

**Table 1:** Cytotoxicity (%) of almond skin samples towards peripheral blood mononuclear cells (PBMC) before and after *in vitro* gastric and gastric plus duodenal digestion. Values represent the means of three experiments ± standard deviations. NS, natural almond skins; NS G, natural almond skins post *in vitro* gastric digestion; NS G+D, natural almond skins post *in vitro* gastric + duodenal digestion; dNG, soluble gastric digesta from natural almond skins; dND, soluble gastric plus duodenal digesta from natural almond skins.

**Inducer**	**500 μg/mL**	**300 μg/mL**	**100 μg/mL**	**60 μg/mL**
NS	89 ± 9.1	40 ± 4.1	8 ± 0.6	0
NS G	91 ± 6.2	50 ± 4.8	10 ± 0.8	0
NS G+D	88 ± 7.8	90 ± 3.1	18 ± 1.1	0
	**100 μL/mL**	**50 μL/mL**	**20 μL/mL**	**10 μL/mL**
d NG	85 ± 5.9	50 ± 3.9	20 ± 0.7	0
d ND	60 ± 4.8	25 ± 1.8	8 ± 0.3	0

**Figure 1:**
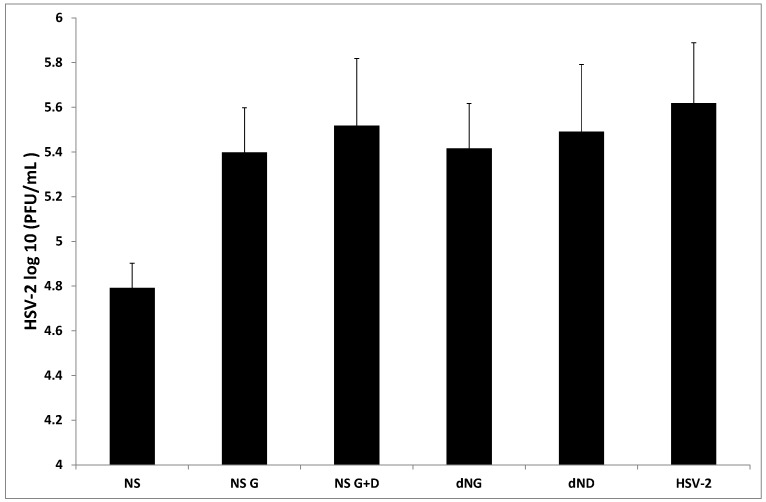
Antiviral activity of almond skin extracts.

As reported in Figure 1, treatment with NS significantly inhibited HSV-2 replication (*p* < 0.05). In the presence of NS (60 μg/mL), PBMC produced 4.79 (±0.11) log_10_PFU/mL, compared with 5.61 ± 0.2 log_10_PFU/mL obtained with untreated PBMC. No inhibition of viral replication was obtained after treatment with NS G and NS G+D, dNG and dND. These data confirmed previous reports, in which the effect obtained with NS has been suggested to be due to the higher concentration of polyphenols compared to all other extracts.

NS, natural almond skins; NS G, natural almond skins post *in vitro* gastric digestion; NS G+D, natural almond skins post *in vitro* gastric + duodenal digestion; dNG, soluble gastric digesta from natural almond skins; dND, soluble gastric plus duodenal digesta from natural almond skins.

Table 2 reports the production of IL-17, IFN-γ, IFN-α and IL-4 by PBMC infected or not with HSV-2. While HSV-2 induced IL-17 and IL-4 production, NS triggered IFN-γ, IFN-α and IL-4, the latter to a higher extent than HSV-2 alone. Furthermore, NS treatment determined a clear-cut production of IL-17 induced by HSV-2, also triggering PBMC to produce marked amounts of IFN-γ, IFN-α and IL-4 in the presence or absence of HSV-2 infection. The viral infection determined a down-regulation of all the cytokines tested. These data further supported that the immunomodulatory effect of NS may be due to the polyphenols present in almond skins, as NS has previously been shown to contain the highest amount compared to the other extracts [2]. Since NS G, NS G+D, dNG and dND did not show any antiviral activity, these fractions were not evaluated in subsequent experiments.

In order to better understand the role of NS on the clear cut-production of IL-17 HSV-2 induced, which seemed to be correlated with the antiviral activity of NS, we have investigated the possible correlation between IFN-γ, IFN-α, IL-4 and IL-17 production. The effect of monoclonal antibodies on production of IL-17 is reported in Table 3A. Neutralization of IL-4 resulted in an unexpected increase of IL-17 in HSV-2, NS and NS + HSV-2, suggesting that IL-4 inhibits IL-17. Except for HSV-2 alone, no significant effects were observed after neutralization of IFN-γ. A significant increase in IL-17 production was observed after neutralization of IFN-α induced by NS, both in the presence or absence of HSV-2. As the neutralization of both IFN-α and IL-4 resulted in an increase in IL-17 production, it is possible to conclude that IL-4 and IFN-α were able to inhibit IL-17.

**Table 2:** Production of cytokines (pg/mL) by peripheral blood mononuclear cells (PBMC) at 48h post natural almond skin (NS) treatment, with and without HSV-2 infection. Values are expressed as the means of four experiments ± standard deviations. NS (60 μg/mL), natural almond skins; NS G (60 μg/mL), natural almond skins post *in vitro* gastric digestion; NS G+D (60 μg/mL), natural almond skins post *in vitro* gastric + duodenal digestion; dNG (10 μL/mL), soluble gastric digesta from natural almond skins; dND (10 μL/mL), soluble gastric plus duodenal digesta from natural almond skins.

	IL-17		IFN-γ		IFN-α		IL-4	
	PBMC	+HSV-2	PBMC	+HSV-2	PBMC	+HSV-2	PBMC	+HSV-2
NS	<15	<15 ^a^	1961 ± 257	2123 ± 380	98 ± 7.2	53 ± 3.8 ^a,b^	325 ± 69	298 ± 43
NS G	<15	41 ± 3.8 ^b^	<8	<8	<3.1	<3.1	69 ± 31	42 ± 12
NS G+D	<15	45 ± 4.3 ^b^	<8	<8	<3.1	<3.1	98 ± 9	52 ± 7 ^b^
dNG	<15	29 ± 3.5 ^a,b^	163 ± 91	102 ± 96 ^a^	<3.1	<3.1	54 ± 26	83 ± 21
dND	<15	39 ± 4.9 ^b^	189 ± 83	<8 ^b^	<3.1	<3.1	41 ± 34	39 ± 8.4
HSV-2		48 ± 4.1		<8		<3.1		181 ± 43
None	<15	<15	<8	<8	<3.1	<3.1	<10	<10

^a^: significantly different (*p* < 0.05) compared with HSV-2 infected PBMC; ^b^: significantly different (*p* < 0.05) compared with uninfected PBMC treated with the same compound.

Production of IL-17 was not significantly affected by addition of recombinant human (rh) IL-4 or rh IFN-γ in HSV-2, but significantly decreased after addition of rh IFN-α (Table 3B). In all cases, production of IL-17 effectively ceased after addition of both rh IFN-α and rh IL-4.

**Table 3:** Effect of varying treatments on IL-17 production (pg/mL) at 48h post NS treatment by peripheral blood mononuclear cells (PBMC) and under the effect of HSV-2 infection. Values are expressed as the means of four experiments ± standard deviations. NS, natural almond skins. nd—not detected. (**A**) Effect of monoclonal antibodies; (**B**) Effect of recombinant human (rh) IL-4, IFN-γ and IFN-α.

**A**	**PBMC**	**+ mAbvs IL-4**	**+ mAbvsIFN-γ**	**+ mAbvsIFN-α**	**+ mAbvsIFN-α and IL-4**
HSV-2	48 ± 4.1	86 ± 14.1	nd	nd	nd
NS	<15	41 ± 3.6	<15	116 ± 18.3	207 ± 25.9
NS + HSV-2	<15	54 ± 5.1	<15	167 ± 20.9	309 ± 41.3
**B**	**PBMC**	**+ rh IL-4**	**+ rh IFN-γ**	**+ rh IFN-α**	**+ rh IFN-α and rh IL-4**
HSV-2	48 ± 4.1	38 ± 5.5	51 ± 7.7	19 ± 2.1	<15
NS	<15	<15	<15	<15	<15
NS + HSV-2	<15	<15	<15	<15	<15

## 3. Experimental Section

### 3.1. General Information

The cytotoxicity test was performed on PBMC. Natural almond skin extracts were diluted in apyrogenic sterile water at the concentration of 1 mg·mL^−1^. To determine the effect of various concentrations on cells viability, the colorimetric assay described by Mosmann [7] was used. HSV-type 2 strain G was used throughout the study. HSV-2 infection was propagated on WISH cell lines. Viral stocks were prepared by pelleting infected cells exhibiting cytopathic effect, and freezing aliquots at −80 °C. Viral titer was assessed on WISH cells and expressed as plaque forming unit (PFU) per mL.

### 3.2. Isolation of Human Peripheral Blood Mononuclear Cells (PBMC)

PBMC were isolated from freshly collected buffy coats of healthy blood donors (Centro Trasfusionale, Policlinico Universitario ‘G. Martino’, Messina, Italy), after centrifugation over Ficoll-Hypaque gradient. PBMC were then washed three times in RPMI 1640 medium (Sigma, Milan, Italy) and cultured at 37 °C under 5% CO_2_ in 24-well plates at a concentration of 2 × 10^6^ cells·mL^−1^ per well. TheRPMI 1640 medium was supplemented with 50 μg·mL^−1^ gentamicin and 5% fetal calf serum (FCS, Sigma).

Treatment using natural almond skin (NS), NS post *in vitro* gastric (NS G), and NS post *in vitro* gastric plus duodenal (NS G+D) digestion at the concentration of 60 μg/mL, as well as soluble gastric (dNG) and duodenal (dND) digesta from NS at the concentration of 10 μL/mL, was previously reported [1]. PBMCs were treated with each of the above described samples infected with HSV-2 strain G at a multiplicity of infection (MOI) 0.1, and incubated for 24 h at 37 °C under 5% CO_2_. The presence of IL-17, IFN-γ, IFN-α, and IL-4 cytokines was determined using an immunoenzymatic method (ELISA, DRG diagnostic, Milan, Italy). The limits of detection were 15, 8, 3.1 and 10 pg·mL^−1^, respectively.

Monoclonal anti-human IFN-γ antibody [mAbvsIFN-γ] was used at the concentration of 0.02 μg/mL, monoclonal anti-human IFN-α antibody [mAbvsIFN-α] was used at the concentration of 0.3 μg/mL and monoclonal anti-human IL-4 antibody [mAbvsIL-4] was used at the concentration of 0.3 μg/mL. All reagents were purchased from DRG Diagnostics (Milan, Italy).

Results are expressed as the means of three experiments ± standard deviation (S.D.). Data were analysed by one-way analysis of variance (ANOVA) and the Student-Newman-Keuls test. Differences were considered statistically significant for *p* value < 0.05.

## 4. Conclusions

It has been shown that herpes viruses can indirectly counteract the phagocyte functions, namely by viral mechanisms of mimicry of cytokines and cytokine receptors [8,9,10]. This highlights the need for the development of new antiviral drugs that seek to combine immunotherapeutic intervention, as an adjunct to antiviral activity, thus conferring added benefit by controlling viral replication. In this work we have demonstrated that HSV-2 was able to induce production of IL-17 and IL-4. Neutralization of both IFN-α and IL-4 determined a marked up-regulation of IL-17, whereas recombinant of both INF-α and IL-4 determined a clear cut-production of IL-17. In conclusion, NS treatment was able to inhibit IL-17 production up-regulating IFN-α and IL-4. Therefore, NS could play a key role for the antiviral activity of almond skins since it is a good inducer of IFN-α.

## Abbreviations

IL: interleukin
IFN: interferon
